# PMCA screening of retropharyngeal lymph nodes in white-tailed deer and comparisons with ELISA and IHC

**DOI:** 10.1038/s41598-023-47105-9

**Published:** 2023-11-17

**Authors:** Rebeca Benavente, J. Hunter Reed, Mitch Lockwood, Rodrigo Morales

**Affiliations:** 1 Department of Neurology, McGovern Medical School, The University of Texas Health Science Center at Houston, Houston, TX USA; 2 Texas Parks and Wildlife Department, Kerrville, TX USA; 3 Centro Integrativo de Biologia y Quimica Aplicada (CIBQA), Universidad Bernardo O’Higgins, Santiago, Chile

**Keywords:** Biological techniques, Neuroscience

## Abstract

Chronic wasting disease (CWD) is a prion disease affecting cervids. CWD diagnosis is conducted through enzyme-linked immunosorbent assay (ELISA) and immunohistochemistry (IHC) in retropharyngeal lymph nodes. Unfortunately, these techniques have limited sensitivity against the biomarker (CWD-prions). Two in vitro prion amplification techniques, real-time quaking-induced conversion (RT-QuIC) and protein misfolding cyclic amplification (PMCA), have shown promise in detecting CWD-prions in tissues and bodily fluids. Recent studies have demonstrated that RT-QuIC yields similar results compared to ELISA and IHC. Here, we analyzed 1003 retropharyngeal lymph nodes (RPLNs) from Texas white-tailed deer. PMCA detected CWD at a higher rate compared to ELISA/IHC, identified different prion strains, and revealed the presence of CWD-prions in places with no previous history. These findings suggest that PMCA exhibits greater sensitivity than current standard techniques and could be valuable for rapid and strain-specific CWD detection.

## Introduction

Chronic wasting disease (CWD) is a transmissible spongiform encephalopathy (TSE) affecting deer, moose, elk, and other related animal species^1,2^. Similar to other TSEs, CWD is characterized by the misfolding of the cellular prion protein (PrP^C^) into a β-sheet rich conformer (PrP^Sc^)^3,4^. PrP^Sc^ particles aggregate and accumulate in the brain causing multiple brain anomalies that invariably lead to death^5^. CWD naturally affects multiple species of cervids, both in free-ranging and captive settings^6^. Several studies have stablished a relationship between the environment and the spread of CWD^7–10^. Deer can shed prions to the environment through saliva, urine and feces^11–15^. In addition, prions can attach to different environmental surfaces and materials including plants, soils, and other relevant surfaces^16–21^ and maintain their infectivity. In the United States, the first case of this disease was detected in 1967 in a captive mule deer. Since then, CWD has been found in 31 states^22^, and some captive facilities have reported CWD prevalence as high as ~ 30%^23^. In addition, CWD infected animals have also been identified in multiple free-range cervid populations in Scandinavia^24–26^ and Korean captive facilities^27^.

Deer farming and hunting has relevant impacts in the United States and particularly in Texas. A study performed in 2022 revealed that the deer industry contributed with $4.3 billion to Texas economy, generating thousands of jobs especially in rural areas (https://nri.tamu.edu/media/3702/economic-values-of-white-tailed-deer-in-texas-2022-survey-part-i.pdf). Unfortunately, the presence of CWD generates relevant negative impacts at both wildlife and economic fronts^28,29^. To address these problems, federal and state agencies have dedicated significant resources to identify potentially infected deer populations and fund management and research programs. Although significant advances have been made, CWD spread is not yet controlled and additional efforts, at different fronts, will need to be considered to eradicate this disease. An additional problem involving CWD lies in its still unclear zoonotic potential. The latter acquire relevance as CWD prions from one cervid species are able to infect multiple others, favoring the appearance of multiple prion strains with unknown potentials to infect other (non-cervid) animals^30,31^.

The first case of CWD in Texas was detected on a free-ranging mule deer in 2012 at Hudspeth county. At present, a total of 21 Texas counties have documented CWD-infected cervids^32^. As mentioned above, one of the most prevalent cervids species in Texas involve white-tailed deer *(Odocoileus virginianus)* with a population of 5.4 million reported as in October 2021^33^. The first cases of CWD-infected white-tailed deer in Texas were documented between 2012 and 2015 on Northwest Texas as well as in Medina county^32^. Despite this large population, the proportion of reported CWD cases is low, especially in free-range animals. In 2022, only 16 CWD cases in free-ranging mule deer and 5 in white-tailed deer were reported^32^. A higher number of CWD cases, but still in a low proportion (n = 123), were recorded for white-tailed deer in Texan captive facilities^32^. Regardless of this low prevalence, the presence of CWD in Texas brings the concern that cases may continue to increase over time. Additionally, there is a concern that animals carrying this disease are not being detected due to the lack sensitivity of ELISA and immunohistochemistry (IHC), which are the currently approved diagnostic techniques^34,35^. In fact, the lack of accurate, sensitive and low-invasive diagnostic techniques is attributed as one of the most crucial causes to the lack of control of CWD spreading^35^.

The management of CWD in captive and free-range deer is different. In captive facilities, regulations require *post-mortem* CWD testing and restriction of animals’ movements among other measures aiming to reduce CWD spreading^36^. For free-ranging animals, the control and management of the disease is more problematic since exposed populations cannot be readily removed and contaminated environments cannot be easily identified and quarantined. Further, the strategies to monitor the prevalence and distribution of CWD rely mainly on testing harvested animals and targeted removal of animals in infected populations, both of which require marked cooperation from hunters and landowners^37,38^. Currently, the gold standard techniques used for CWD screening include ELISA and IHC of retropharyngeal lymph nodes (RPLN) and obex^35^. Nonetheless, the sensitivity of both techniques is limited^34,39–41^. Recently, two promising in vitro prion amplification techniques have gained attention as possible diagnostic tests for CWD. The first include the real-time quaking-induced conversion (RT-QuIC). This technique uses soluble recombinant prion protein that is induced to aggregate in the presence of CWD prion particles in a given sample. In RT-QuIC, the readouts are provided as thioflavin T fluorescence in real time and this assay is usually completed between 24 and 90 h^42–49^. Considering the latter, and the fact that RT-QuIC products are not infectious, this technique presents itself as a cheap, fast and safe alternative to diagnose CWD^50^. RT-QuIC has been used to detect CWD prions in multiple tissues and fluids from deer, including brain, feces, cerebrospinal fluid, RPLN, among others^51–56^. Due to its potential for high-throughput, two recent publications have reported the potential feasibility of RT-QuIC for CWD detection using RPLN. For that purpose, these studies compared RT-QuIC performance with the currently approved diagnostic methods. In the first study^57^, RPLN from 1300 deer were analyzed. This study showed that RT-QuIC was capable of detecting 178 CWD-positive animals, while IHC and ELISA identified 176 and 184, respectively. In summary, this study showed a diagnostic agreement of 98% or above between the three techniques^57^. In a second study, RPLN from 519 animals were analyzed^58^. There, 13 were considered CWD-positive by ELISA and confirmed by IHC, and 11 of them were further confirmed by RT-QuIC^58^. Unfortunately, these results suggest that RT-QuIC present a similar diagnostic power in RPLN when compared with the currently used diagnostic techniques.

A second prion replication method, termed protein misfolding cyclic amplification (PMCA), utilizes brain homogenates of healthy animals (usually transgenic mice) as a substrate for conversion and replication of PrP^Sc^ in a given sample. After cyclic rounds of incubation and sonication, PMCA products are visualized by western blot after proteinase K (PK) treatment^59^. Differently from RT-QuIC, PMCA generates infectious materials that can be used to study biological aspects of prion biology^60,61^. Importantly, PMCA faithfully reproduces the strain-specific characteristics of the disease-associated prions (PrP^Sc^), which is relevant for both basic research and diagnostic purposes^62–64^. PMCA has been tested as a potential diagnostic tool in multiple samples derived from CWD infected cervids, including saliva, urine, brain, semen, blood, RPLN, obex and several other tissues and fluids^65–70^. The sensitivity of both PMCA and RT-QuIC seems to be comparable according to multiple studies^34,54^; however, reports comparing the diagnostic value of both techniques in the same sample type and animal cohorts are still pending. Importantly, no studies comparing the diagnostic power of PMCA to ELISA and IHC in a large number of RPLN from deer exposed to CWD are available. Here, we studied the CWD-prion detection efficacy of PMCA in RPLNs using a large cohort (n = 1003) of specimens derived from captive and free-ranging white-tailed deer. Results from this PMCA screening were compared with already available data on the same samples using IHC and/or ELISA.

## Results

### Standardization of dot blots for analysis of PMCA products

**Figure 1:**
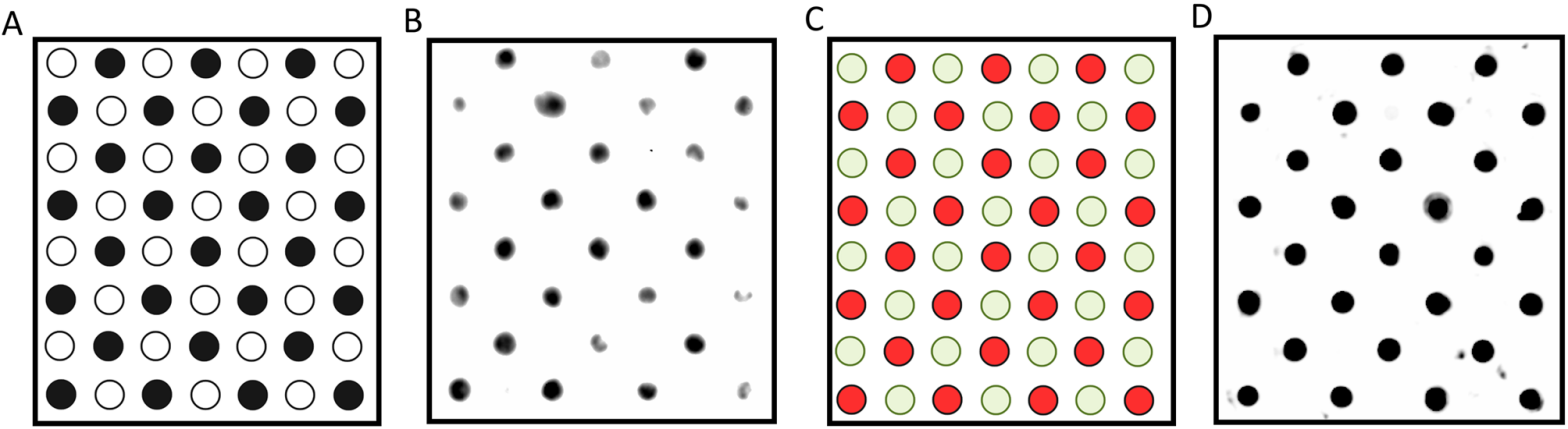
Representative images depicting the visualization of PMCA products by dot blots. (**A**) Scheme depicting the position of PK-digested (white dots) and untreated (black dots) brain extracts from an uninfected (PrP^Sc^-free) Tg1536 mouse. (**B**) Dot blot depicting PK-digested and untreated brain extracts from an uninfected (PrP^Sc^-free) Tg1536 mouse placed in a membrane following the scheme shown in (**A**). (**C**) Scheme depicting the position of CWD-seeded (red dots) and unseeded (green dots) PMCA products. (**D**) Dot blot results from CWD-seeded and unseeded PMCA products following the plan shown in (**C**). All samples in C were PK treated as explained in Materials and Methods. The CWD prion “seed” used in this experiment correspond to the brain extract of a terminally ill tg1536 mouse (expressing the deer prion protein). This mouse was intracerebrally inoculated with the brain extract of a terminally ill, experimentally infected white-tailed deer (PrP 96GG, kindly donated by Dr. Edward Hoover).

One of the disadvantages of the PMCA technique includes the time-consuming visualization of products by western blot. To overcome this limitation, reduce the amount of sample needed for analysis, and increase throughput, we standardized dot blots for the analysis of the PMCA products. This is convenient considering the analysis of large animal cohorts as the one tested in this study (n = 1003). To achieve this, we checked dot blots for their specificity to discriminate PrP^Sc^-positive from PrP^Sc^-negative samples. In a first approach, we analyzed brains from healthy Tg1536 mice^71^ (overexpressing deer prion protein) with and without PK treatment. As shown in Fig. 1A and B, PrP^C^ was easily detected by this technique and signals completely disappeared after PK treatments. Then, we evaluated the discrimination of PMCA products derived from PrP^Sc^-seeded and unseeded reactions. Our results further confirmed the specificity of our dot blot system as all seeded PMCA reactions provided positive signals while unseeded samples were not visualized (Fig. 1C and D).

**Figure 2:**
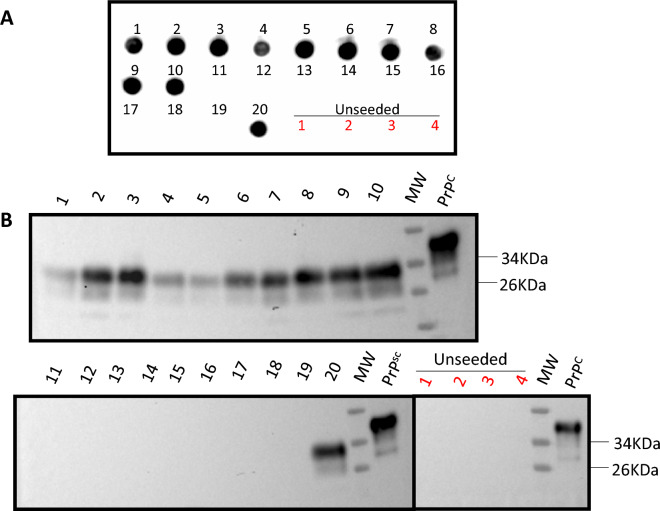
Confirmation of dot blot signals for PMCA products using western blots. Evaluation of PMCA products from 20 selected RPLNs by dot blots (**A**) and western blots (**B**). Numbers 1–20 were arbitrarily assigned and used to compare outcomes in both techniques. Four unseeded reactions (1–4, in red) are shown to demonstrate the typical outcomes observed in our negative controls. All samples were PK digested, with the exception of “PrP^C^” in (**B**) that is used as a control of electrophoretic mobility and antibody reactivity. All membranes where probed with the 6D11 antibody. Numbers at the left of blots in panel (**B**) depict molecular weight markers. MW: molecular weight marker. ﻿Raw data is provided as Supplementary Information.

Regardless of the specificities observed in our dot blot system, we understand that the chances to obtain false positive results increase using this technique. In addition, dot blots do not allow the visualization of the classical electrophoretic mobility shift observed between PK -treated and -untreated samples that help to confirm the presence of PrP^Sc^. For that reason, all dot blot -positive PMCA products obtained from deer’s RPLNs reactions were further confirmed for their PrP^Sc^ content using western blots. As observed in Fig. 2, western blot revealed the presence of PrP^Sc^ electrophoretic patterns in all PMCA positive samples, including positive controls and specimens tested as part of this screening. These results show that our PMCA system coupled with a dot blot protocol can effectively discriminate PrP^Sc^-containing samples from CWD-free specimens.

### Screening of a large cohort of RPLN specimens reveal the increased sensitivity of PMCA over ELISA and IHC

**Figure 3:**
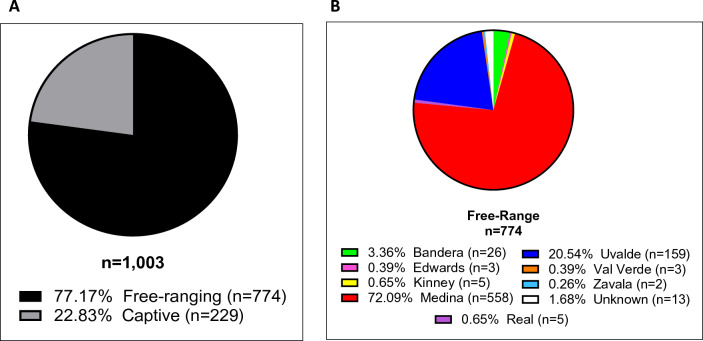
Distribution and origin of the white-tailed deer RPLN samples analyzed. (**A**) Distribution of samples considering their habitats (captive or free-ranging). (**B**) Distribution of free-ranging samples by Texas county.

**Figure 4:**
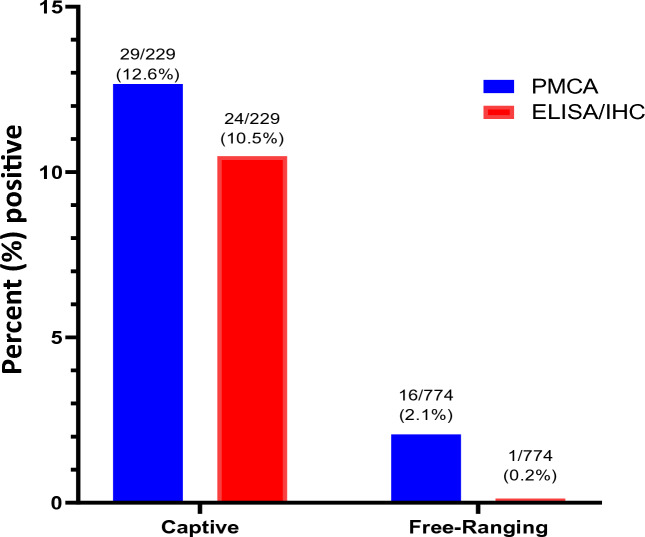
Summary of the PMCA-screening in 229 RPLN specimens collected from captive and free-ranging Texas white-tailed deer. Comparison of CWD status for RPLN specimens in captive and free-ranging deer by IHC/ELISA (red) and PMCA (blue). The ratios depicted at the top of each column represent the number of animals identified as CWD-prions carriers by each technique versus the total number of animals analyzed. Percentual values of detection by each technique are also provided.

**Figure 5:**
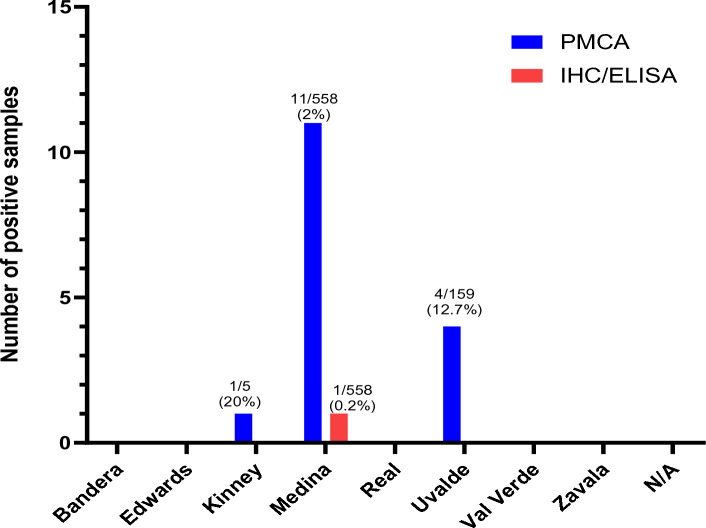
Summary of the PMCA-screening in 774 RPLN specimens collected from free-ranging Texas white-tailed deer by county. Distribution of PMCA (blue) and IHC/ELISA (red) results for free-ranging white-tailed deer by Texas counties. The ratios depicted at the top of each column represent the number of animals identified as CWD-prions carriers by each technique versus the total number of animals analyzed. Percentual values of detection by each technique are also provided. *N/A* not assigned—unknown county.

Considering the effectiveness of dot blots in assessing PrP^Sc^ signals and the possibility to increase the throughput of PMCA screenings, we analyzed RPLNs from 1003 white-tailed deer collected in Texas. These samples were obtained from a depopulated captive facility (n = 229) and from free-ranging deer at different Texas counties (n = 774) (Fig. 3). All tested samples were previously evaluated by IHC and/or ELISA. Importantly, the CWD-status of these samples were available to the researchers running the PMCA assay only after the screening was completed. The overall detection in captive and free-ranging samples by ELISA/IHC and PMCA is presented in Figs. 4 and 5. In the case of captive deer, we were able to detect the presence of CWD-prions in 12.6% of the samples (29/229) while ELISA/IHC provided positive results in 10.5% specimens (24/229) (Fig. 4). This show a superior, although modest, increase in detection for PMCA when compared with the currently approved diagnostic methods.

The increased CWD-prion detection potential of PMCA over ELISA/IHC was best appreciated in samples collected from free-ranging animals. There, PMCA identified 16 samples as CWD-positive, a higher number compared to the single positive specimen detected by the other diagnostic methods. It is important to note that most of the free-ranging positive samples were obtained from two counties with mandatory CWD testing requirements: Medina (n = 11) and Uvalde (n = 4) (Fig. 5). Both counties had CWD containment and surveillance zones established in response to detections of CWD in free-raging and captive animals. It is relevant to note that our PMCA screening identified a CWD-positive deer in Kinney county (Fig. 5) where no cases of CWD have been reported to date. However, due to the uneven number of samples tested in each county, the results of this screening cannot be used as indicators of CWD-positivity across counties.

It is important to mention that while screening the captive and free-ranging-derived samples, only one of the specimens (captive) providing positive results in ELISA/IHC was negative in PMCA. This is relevant, as these results support the specificity of our assay. The single sample providing positive ELISA/IHC results but negative outcomes by PMCA was tested three additional times after CWD-statuses were unveiled to the investigators. PMCA outcomes for this sample were maintained in all replicates. Additionally, and as mentioned previously, all PMCA samples that resulted in positive detection in dot blots were further confirmed for their PrP^Sc^ content by western blots. In this study, all samples displaying positive signals in dot blots displayed the expected electrophoretic mobility shift after PK treatments (Fig. 2). Overall, these results demonstrate the increased CWD-prion detection potential of PMCA over IHC/ELISA, and likely RT-QuIC, in RPLN specimens of white-tailed deer. These results are summarized in Table 1.

**Table 1:** Comparison of IHC/ELISA and PMCA CWD-prion detection considering the habitat of white-tailed deer included in this study.

White-tailed deer habitat	Technique	Detection ratio (detected/total) samples	Fold change
Free-ranging	IHC/ELISA	1/774	15.99
	PMCA	16/774	
Captive	IHC/ELISA	24/229	1.21
	PMCA	29/229	

Fold-change increases in PMCA detection over IHC/ELISA is depicted in the right-end column.

### Detection of different prion strains in the state of Texas

**Figure 6:**
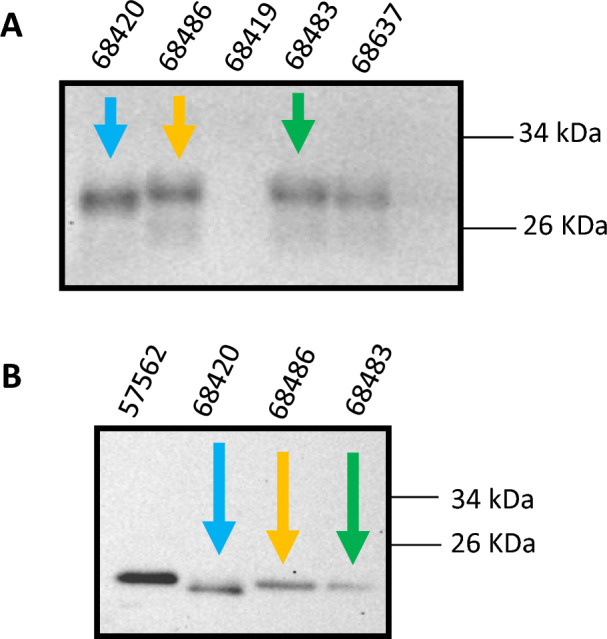
Electrophoretic mobility differences of selected PMCA products derived from RPLNs of free-ranging Texas white-tailed deer. (**A**) Electrophoretic mobilities of PMCA products from four positive and one negative PMCA products. (**B**) Electrophoretic mobilities of PMCA products from four selected positive PMCA products after PNGase treatment. Membranes were probed with the 6D11 antibody. All samples shown in this figure have been PK-treated. Numbers at the top of each panel are arbitrary and depict identification codes. Colored arrows indicate the same samples in both panels. Numbers at the left of blots depict molecular weight markers. Raw data is provided as Supplementary Information.

Prions can manifest in multiple strains, each one linked with specific molecular, pathological and infective properties^72,73^. Notably, multiple CWD strains have already been identified in naturally occurring cervid populations of North America and Europe^74–77^. The identification and study of CWD strains in nature is of capital importance to understand their effect within animal populations, and their interactions with the environment and other animal species. Differently from RT-QuIC, PMCA is able to faithfully propagate the molecular features of prion strains while maintaining their unique infectious properties^78,79^. In this study, we were able to discriminate two different electrophoretical mobilities in positive PMCA products (Fig. 6). Specifically, sample 68420 (blue arrow) from Medina county presented a lower electrophoretical mobility compared to other samples identified as CWD-positive in our screening (yellow and green arrows). This result was confirmed by running PMCA products after deglycosylation (facilitating the identification of changes in the single, unglycosylated PrP band). Importantly, the sample displaying this unique electrophoretic mobility (indicative of a different protein conformation, hence, a different prion strain) was obtained from a free-ranging animal. This suggests that different CWD prion strains are naturally present in wild Texas deer populations.

## Discussion

Currently approved diagnostic methods for CWD include ELISA and IHC. These assays are usually applied to obex and lymph node tissues collected *postmortem*^36^. Multiple reports describe potential strategies to diagnose live animals using less invasive samples such as blood, urine, feces, saliva and skin^15,52,80–85^. In all these studies, the diagnostic sensitivity is established by using the CWD-status information obtained *postmortem* through the above-mentioned approved methods. Considering the theoretical higher sensitivity of prion replication assays over ELISA and IHC, it is imperative to study the performance of these amplification techniques in previously tested lymph node tissues. At present, two studies comparing the efficacy of RT-QuIC to identify CWD-seeding activity in lymph node tissues from white-tailed deer have been communicated^57,58^. These studies reported similar results when using both set of techniques, preliminarily suggesting that RT-QuIC has no major advantages compared to ELISA/IHC for this specific sample type. These unexpected results could be caused by the composition of the biological matrix, as other biological samples have been shown to alter PMCA and RT-QuIC efficiencies^66,86^. Importantly, some reports suggest that PMCA and RT-QuIC have differential performances depending on the type of sample being studied^66,82,83,86^. At present, the PMCA screening of white-tailed deer lymph node tissues collected from large cohorts has not been reported. To fill this gap in knowledge, we screened over 1000 RPLN specimens from free-ranging and captive white-tailed deer that were previously tested using ELISA/IHC.

Our study included 774 RPLN samples from free-ranging white-tailed deer, and 229 equivalent specimens from captive animals. In both cases, PMCA provided higher prion detection ratios compared with ELISA/IHC. Although the increase in detection was modest (20.83%) in captive animals, the identification of CWD-positive deer in free-ranging animals was considerably higher (approximately 16 folds). We believe that these differences are due to the uneven exposure of CWD-prions for the animals in these two groups. Due to the animal density of deer in captive facilities, their exposure to the agent is expected to occur in both higher quantities and frequencies that will favor faster peripheral replication and tropism to lymphoid tissues. In natural environments, the exposure of CWD prions is expected to occur infrequently and in lower doses, increasing the times between prion exposure and clinical disease. The latter is expected to be linked with a slower progression in peripheral prion accumulation, a fact that will explain the lower diagnostic efficiency of ELISA and IHC. Although reasonable, these hypotheses will require further evaluation in different captive and free-ranging cohorts of animals, and using different prion detection methods.

Although our results suggest a superior performance of PMCA over RT-QuIC in RPLN screenings, several technical limitations still persisted. One of the main disadvantages of PMCA over RT-QuIC is the time that the former method takes to provide results^59^. One of the main sources for these extended processing times lies in the visualization of the PMCA products through western blotting^59^. To partially address this concern, this study included the standardization of dot blots for the visualization of PMCA products. One limitation of dot blots includes their potential to provide positive signals in samples that did not experienced a proper PK digestion. This could result in an increase of the rate of false positive results, compromising the overall detection system. Considering this, our dot blot system was controlled by multiple known positive and negative samples that supported our readings. To add rigor to our methodology, all PMCA products providing positive signals in dot blots were further evaluated by western blotting to visualize the specific electrophoretic mobility of PK digested PrP^79,87^. By using this methodology, no false positive signals were found for all CWD-positive PMCA products. This suggest that the use of dot blots may be useful to reduce processing times when handling a large number of samples. Nevertheless, the potential of false positive using PMCA and RT-QuIC is always a possibility. For that reason, experiments involving these assays need to be performed with caution by trained personnel and using appropriate positive and negative controls. We are confident that the positive results obtained in this screening are not due to contamination for several reasons. First, we used a large number of negative controls (unseeded PMCA reactions) across the experiment (n = 108, over 10% of the samples analyzed) and none of these controls provided positive signals. Second, our extensive experience using PMCA has shown that once a PMCA set is contaminated, a high number of positive reactions arise. This was not observed in this particular screening. Third, all the samples that were positive by IHC and/or ELISA, except for one, were also positive by PMCA (discussed below).

An additional positive outcome of this project includes the identification of different CWD-prion strains. Prions can manifest in different strains with the ability to differentially transmit within and across species^88,89^. For that reason, it is important to recognize and study potential strains operating in natural settings. Examples of the potential repercussions of prion strains lie in studies demonstrating that Norwegian deer and reindeer CWD prions are unable to infect humanized mice^90^. On the contrary, an in vitro study showed the potential of American deer prions to infect humanized mice after serial passaging, as well as other animal species^91^. One advantage of PMCA over RT-QuIC lies in the ability of the former to discriminate between prion strains^78,79^. This proved to be useful in our screening, as PMCA products with different electrophoretic mobilities after PK digestion were identified. Differences in electrophoretic mobility after PK digestion are considered as one of the gold-standard features to discriminate between prion strains. The properties of the different CWD prion strains identified in our screening are currently unknown, and whether they are equivalent to other previously identified CWD agents in white-tailed deer will be addressed in future studies. Current experiments in our laboratory are focused to characterize the identity of this particular CWD prion variants in terms of their biochemical, pathological and infectious properties. Future PMCA screenings in larger animal cohorts collected from geographically diverse sites may unveil the presence of additional, naturally occurring strains operating in captive facilities and in the landscape.

As mentioned above, PMCA was able to identify all but one of the CWD-positive RPLNs specimens detected by IHC. This outcome could be due to multiple reasons, including the following: (i) PMCA is unable to detect specific naturally-occurring prion strains, (ii) the uneven distribution of PrP^Sc^ deposits in this tissue, (iii) the sample provided a false positive signal in IHC/ELISA, or (iv) a sampling error. In order to address the former, this sample is currently being investigated by other prion detection methods, including RT-QuIC and bioassays. Future studies will provide clarity on whether PMCA is able to detect only a limited array of CWD prion isolates.

Although this study provides relevant evidence useful for CWD prion detection, some limitations are also identified. One of them involves the regional limitation of our sample. The incidence of CWD in Texas is low compared with other states^32,92^. Considering this, the samples tested in this study may not be representative of the whole CWD spectrum, and future studies analyzing larger and diverse cohorts may be needed to confirm our results. In addition, this experiment is restricted to white-tailed deer, one of the many animal species affected by CWD^32,92–96^. To address this problem, future studies considering mule deer, elk, and other species should be performed. Finally, other sample types should also be considered in future studies to identify potentially relevant specimens that could be used for diagnostic purposes.

Overall, our study provides relevant information for the detection of CWD prions by comparing established techniques with a prion replication method. As expected, our results demonstrate the higher diagnostic potential of PMCA over the currently approved diagnostic techniques when using the same sample type. Moreover, we provide specific methodologies that may increase diagnostic throughput when screening by PMCA. We believe that the data delivered in this manuscript may be useful for wildlife scientists, deer breeders, regulatory agencies and researchers.

## Materials and methods

### Sample collection and preparation

Retropharyngeal lymph nodes (RPLN) from free-ranging and captive white-tailed deer were donated by hunters or collected by Texas Parks and Wildlife Department (TPWD) personnel from breeding premises. These samples were stored at − 20 °C until shipped to UTHealth facilities where they were also stored at − 20 °C. Each sample was stored in an individual bag for free range samples and in double bags for captive samples. Specimens were partially thawed in a disposable container and a piece of tissue (0.2 g) was collected using disposable forceps and blades. The disposable container, blades and tweezers were replaced after use (in contact with a single sample). In addition, manipulator’s gloves were replaced at least once when handling a single sample. The use of disposable equipment and personal protective equipment (PPE) was critical considering the ultrasensitive nature of PMCA that facilitates cross-contamination. Tissues were homogenized at 20% w/v in PBS containing a cocktail of protease inhibitors (Roche, Basel, Switzerland) using a Precellys 24 dual homogenizer (Bertin instruments, MP biomedicals, Irvine, CA, USA).

### Protein misfolding cyclic amplification (PMCA)

The PMCA process was carried out as reported in detail in our prior publications^59^and optimized for CWD^67^. In short, PMCA substrate was prepared from Tg1536 mice’s brains homogenized at 10% w/v in PMCA conversion buffer (150 mM NaCl, 1% Triton X-100, and a protease inhibitors cocktail (Roche, Basel, Switzerland) in PBS). Ninety µL aliquots of substrate were combined with 10 µL of RPLN homogenates (equivalent to 2 µg of wet tissue) or positive control brain extracts. The first PMCA round consisted of 144 incubation/sonication cycles, while the second and third rounds included 96 cycles each. Serial PMCA rounds were performed by mixing 10 µL of PMCA products with 90 µL of fresh PMCA substrate. Each PMCA cycle consisted of 29 min and 40 s of incubation, and 20 s of sonication. Each PMCA set included reactions supplemented with serial dilutions of a CWD brain of known seeding activity, as described^65^. Negative controls included four unseeded reactions per PMCA set. Results were assessed at the third PMCA round. It is important to consider that all unseeded PMCA reactions included in our assay (n = 108) resulted in negative PrP^Sc^ signals. The positive control PMCA reactions (as shown in Fig. 1) were prepared by mixing PMCA substrates with brain extracts from a terminally ill Tg1536 mouse infected with CWD (10^–5^ dilution).

### Proteinase K (PK) treatment

To visualize the content of the protease-resistant prion protein present in the PMCA products, samples were treated with PK (100 µg/mL final concentration, Sigma-Aldrich, Saint Louis, MO, USA). The conditions used for PK digestion included 37 °C with shaking at 450 rpm (in an Eppendorf thermomixer) for 80 min. PK reactions were stopped by the addition of either phenylmethylsulphonyl fluoride (Sigma-Aldrich, Saint Louis, MO, USA) at a final concentration of 5 mM and heating (70 °C for 10 min) for dot blot analyses, or LDS sample buffer (Fisher Scientific, Walthmam, MA, USA) and heating (95 °C for 10 min) for western blotting.

### Dot blotting

To visualize PK-resistant PrP, 5 µL of PK-digested PMCA products were placed in a nitrocellulose membrane (GE Healthcare Amersham, Chicago, IL, USA) using a Bio-dot apparatus (Bio-Rad Laboratories, Hercules, CA, USA), following manufacturer’s recommendations. The membranes were dried using a hairdryer and placed into an incubation chamber containing 3 M guanidinium hydrochloride for 10 min. The membrane was rinsed using washing buffer (1X PBS and 0.05% Tween20 (Sigma Aldrich, Saint Louis, MO, USA)), then incubated with 5% w/v non-fat milk solution and probed with monoclonal purified 6D11 antibody (Biolegend, San Diego, CA, USA) at 1:10,000 dilution. Membranes were washed using washing buffer (0.05% v/v of Tween20 (Sigma Aldrich, Saint Louis, MO, USA) in PBS) and then incubated with a polyclonal anti-mouse IgG (whole molecule)–peroxidase antibody produced in sheep (Sigma-Aldrich, Saint Louis, MO, USA) at a 1:3000 dilution. Membranes were washed and developed using ECL (GE Healthcare Amersham, Chicago, IL, USA) following manufacturer’s recommendations. Samples providing positive PK-resistant PrP signals were subsequently run by western blot to rule out the presence of false positives.

### Western blotting

For western blots, a similar procedure as dot blot was performed with the difference that PK-digested samples were fractioned in NuPAGE 12% Bis–Tris gels (Invitrogen, Carlsbad, CA, USA) and then transferred to nitrocellulose membranes (GE Healthcare Amersham, Chicago, IL, USA), as previously reported^67,69^. Membranes were blocked using 5% w/v non-fat milk solution and probed with monoclonal purified 6D11 antibody (Biolegend, San Diego, CA, USA) at a 1:10,000 dilution. Besides of the step involving incubation with the guanidinium hydrochloride solution, the rest of the process was the same as described for dot blotting.

### Protein deglycosilation assay

To study the presence of different electrophoretic mobilities in PK-digested PrP^Sc^, positive PMCA products were subjected to deglycosylation with glycerol-free N-glycosidase (New England BioLabs, Ipswich, MA, USA). Briefly, 50 µL of sample were digested with 50 µg/mL of PK (final concentration) for 1 h at 37 °C and then centrifuged at 100,000×*g* for 1 h at 4 °C. The resulting pellets were resuspended in 50 µL of denaturant buffer (New England BioLabs, Ipswich, MA, USA) and heated for 10 min at 95 °C. Then, 6.5 µL of NP40 buffer, 6.5 µL of 10X deglycosilation buffer and 1 µL of Peptide—N-Glycosidase F (PNGase F) solution were added to the sample followed by overnight incubation at 37 °C with shaking at 450 rpm in an Eppendorf thermomixer. Finally, 25 µL of 4X LDS sample buffer were added to the mixture and western blot analysis was performed as described above. The membranes were probed with monoclonal purified 6D11 antibody (Biolegend, San Diego, CA, USA) at a 1:10,000 dilution.

### Supplementary Information

Supplementary Information.

## Data Availability

The datasets used and/or analyzed during the current study available from the corresponding author on reasonable request.
