# Supplementary figures and images for: PMCA screening of retropharyngeal lymph nodes in white-tailed deer and comparisons with ELISA and IHC
Source: Sci Rep. 2023 Nov 17;13:20171. doi: 10.1038/s41598-023-47105-9 (PMC10656533; doi:10.1038/s41598-023-47105-9)

## Slide 1
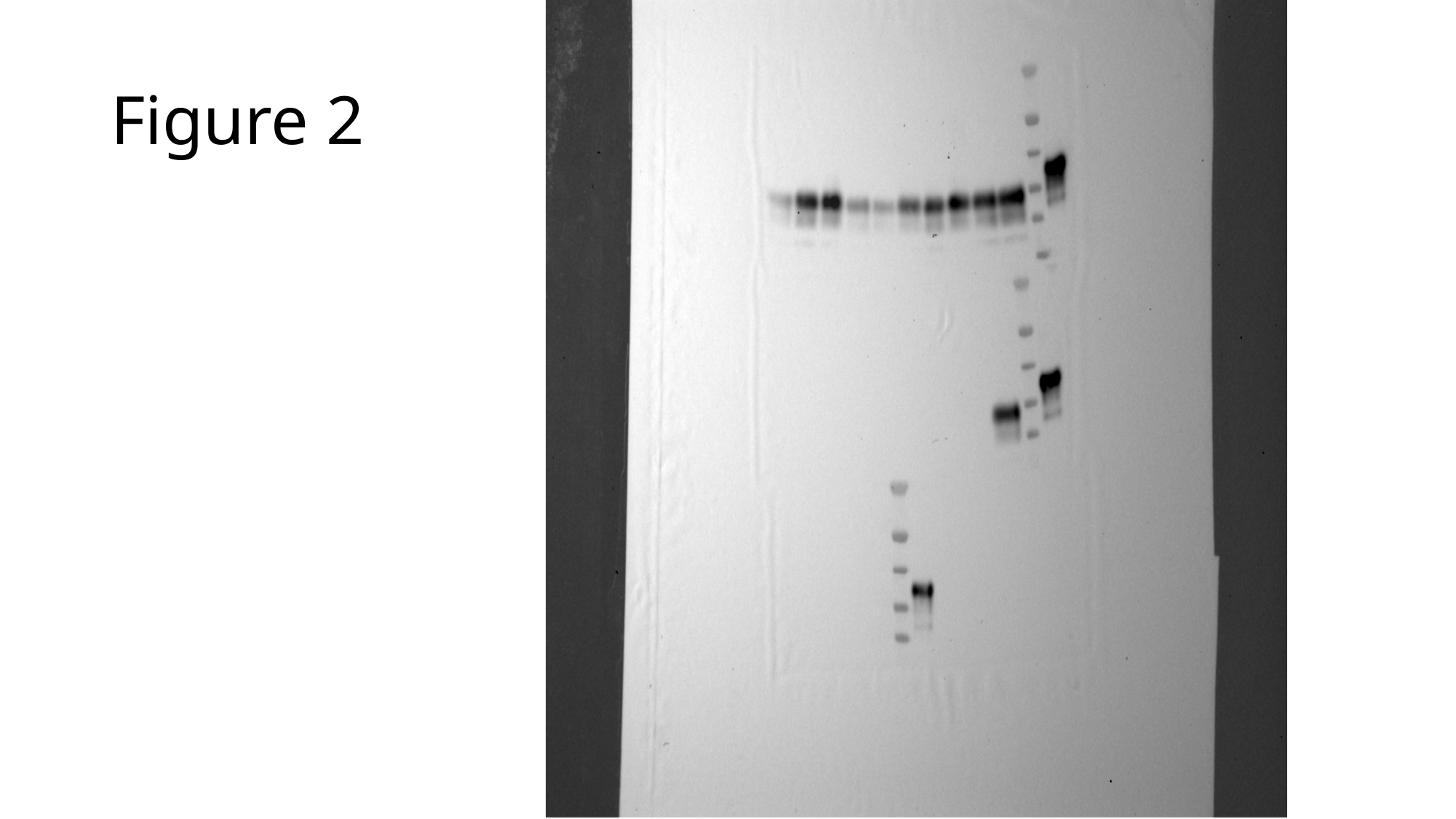

# Figure 2

## Slide 2
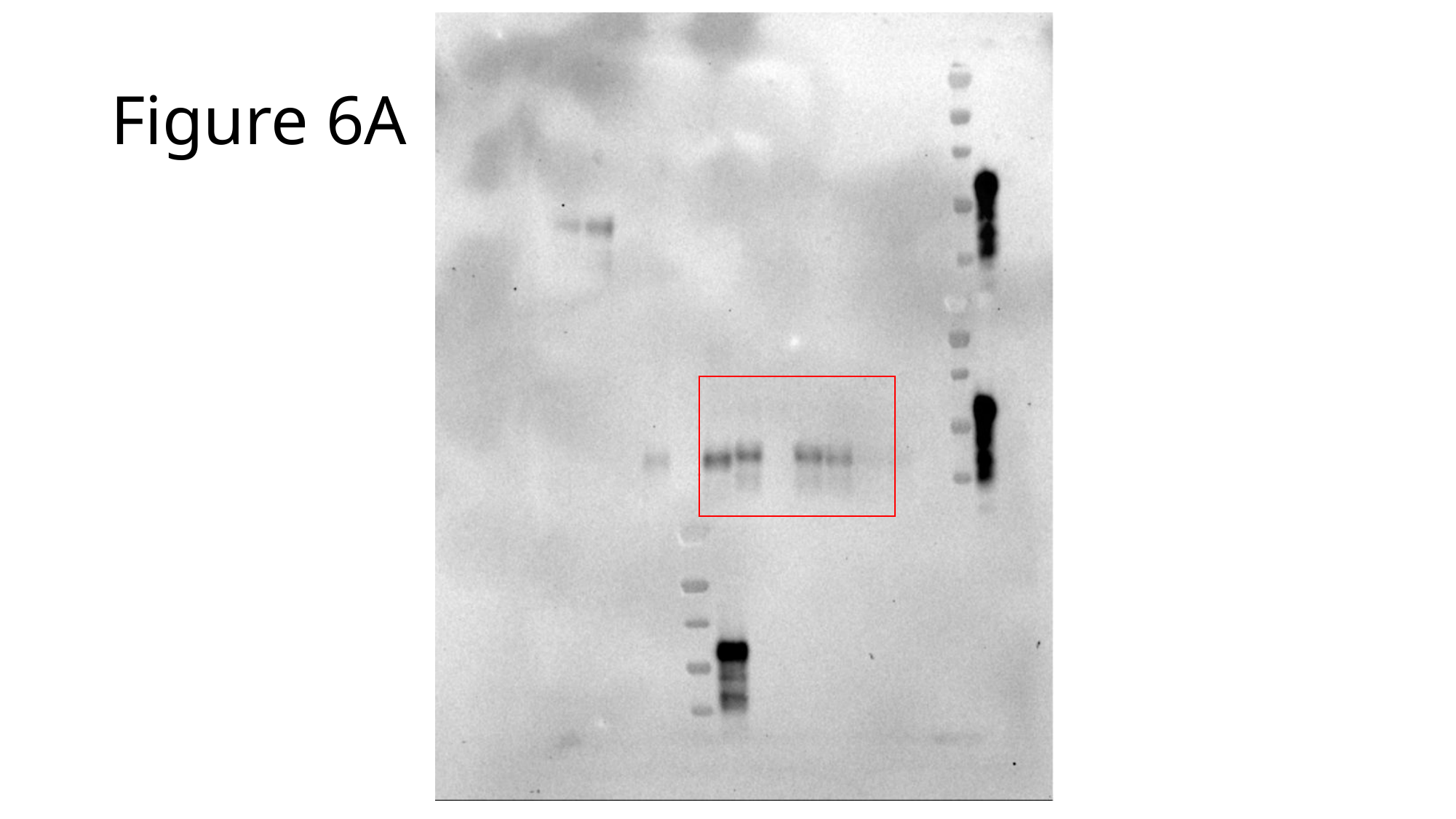

# Figure 6A

## Slide 3
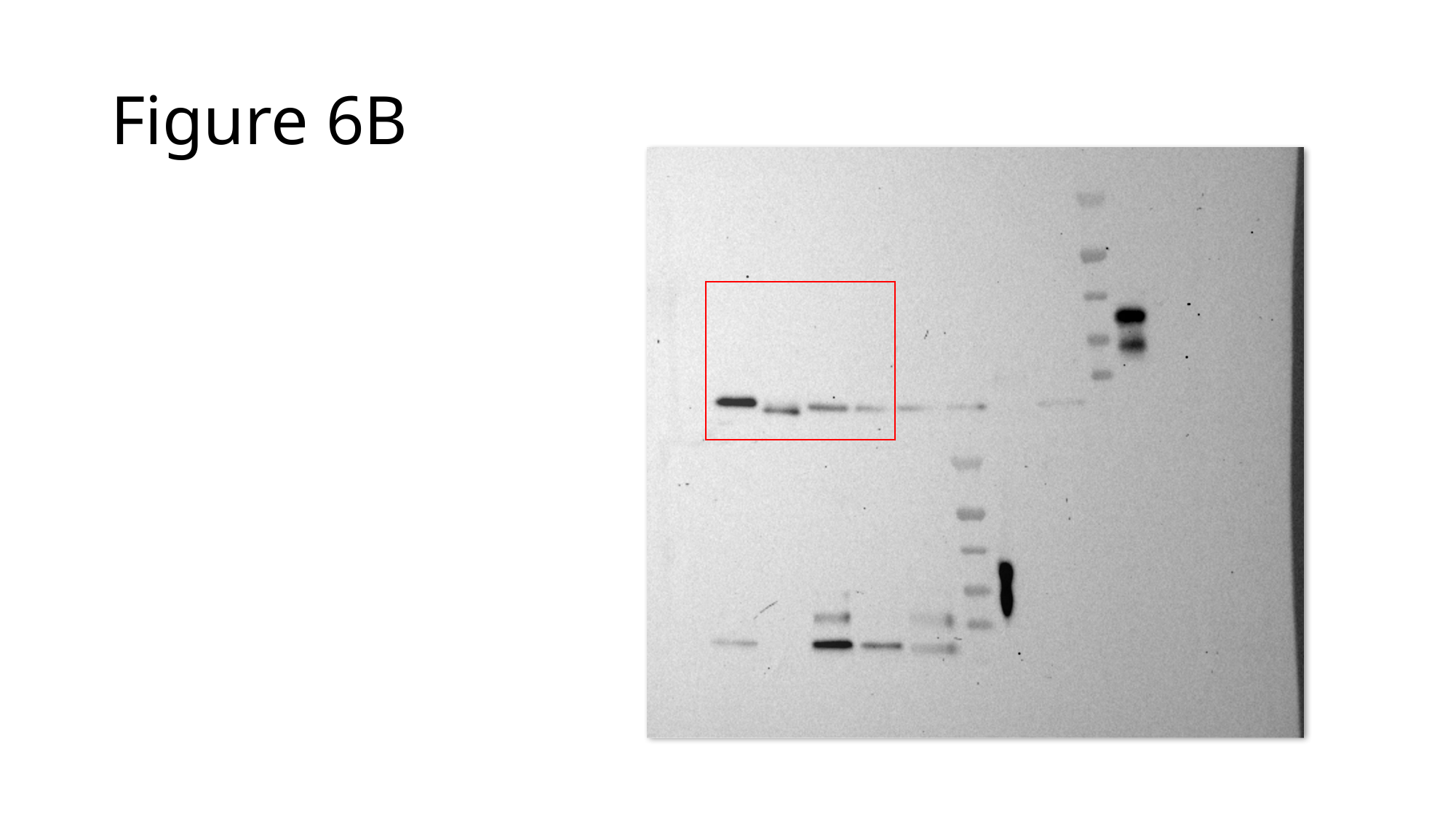

# Figure 6B

Supplement: Supplementary file 1 — Supplementary Information. [file 41598_2023_47105_MOESM1_ESM.pptx]
